# Thoughts on the Condensation of Gold Fillings

**Published:** 1857-06

**Authors:** W. Muir Rogers


### THOUGHTS ON THE CONDENSATION OF GOLD FILLINGS.

Much is said in the journals about the liability of crystal gold to crumble in the mouth. I have not tried it, but I have used the annealed foil with success, and I find that *too much* condensation may be made with serrated instruments, causing the filling to crumble. May not the failures with crystal gold be attributable to the fact that experimenters with it apply the serrated points too often over the same surface, thus tearing the prominences already made on the filling from their position so often as to make them brittle and also incapable of adhesion in any new contact into which they may be forced. Of course, we bear in mind, with reference to this remark, that the point of the condenser is a collection of small wedges, which are forced into the gold at each application, without reference to the exact position in which they have before penetrated. I have frequently noticed that large fillings, put in by me in the ordinary way, with either strips or cylinders, and condensed for a long time upon the surface with *serrated* instruments, will *crumble* rather than *cut*, as I take away the protruding gold to finish, just as deep as I suppose the points have penetrated, while the surface below this is tough and adhesive, and cuts away apparently in solid shavings. A gold bar too often bent upon itself, will become brittle, and finally its particles will separate from their molton contact; and surely it is no objection to crystal gold, that it possesses this same property, so common to gold in any of its forms. Too little attention to condensation must, of course, end in the same result—the difference being that in the one case adhesion is not procured, and the other, the adhesion at first secured is afterwards destroyed.

Pardon me if the above suggestion is too trivial to be obtruded upon your notice, and believe me in haste, but respectfully yours, W. MUIR ROGERS.

